# Virtual reality aggression assessment with social interaction: early evidence for validity from two pilot studies

**DOI:** 10.3389/fpsyg.2025.1585609

**Published:** 2025-06-13

**Authors:** F. Sappelli, J. Lobbestael, D. L. van Haalen, I. Böckmann, B. E. Bulten, R. J. Verkes

**Affiliations:** ^1^Department of Diagnostics, Research, and Education, Pompestichting Forensic Psychiatry, Nijmegen, Netherlands; ^2^Department of Psychiatry, Radboud University Medical Center, Nijmegen, Netherlands; ^3^Faculty of Psychology and Neuroscience, Maastricht University, Maastricht, Netherlands

**Keywords:** aggression assessment, aggression paradigm, ecological validity, immersive scenarios, social interaction, virtual reality

## Abstract

**Introduction:**

Assessment of aggression proneness is important for clinical practice and research. Virtual Reality (VR) is a promising technology for aggression assessment because of the possibility of creating scenarios with ecological and external validity and experimental control, potentially overcoming limitations of traditional methods like self-report trait questionnaires, observation surveys, and laboratory paradigms.

**Methods:**

This explorative study investigated a VR scenario in a pilot with aggressive patients (*n* = 12) and a follow-up with students (*n* = 12). The VR scenario consisted of social interactive roleplays with virtual characters (avatars). It consisted of a neutral scene, an instruction scene and two provocative scenes: one with a socially and verbally aggressive, uncooperative female avatar (Provocation 1) and one with a dominant, unreasonable, intimidating male avatar (Provocation 2). The primary outcome was VR-displayed aggression, assessed with a VR-customized version of the Social Aggression and Dysfunction Questionnaire. Lifetime aggression self-report scales were also administered.

**Results:**

The main analysis revealed that both patients and students showed higher levels of aggression in provocative scenes than in neutral and instructional scenes. Exploratory analyses suggested that patients displayed increased aggression in the second provocation compared to the first, while no such difference was observed among students. Comparatively, patients showed more aggression than students in Provocation 2, but not in Provocation 1. Positive moderate correlations were found between VR-displayed aggression and trait questionnaires.

**Discussion:**

The current study shows that aggressive behavior can be evoked with our VR aggression assessment scenario and that the level of aggression can be systematically assessed using a standardized aggression observation scale. Explorative results imply that the VR scenario has construct, known-group and concurrent validity. The results also imply that VR is potentially closing the correlational gap between behavioral tasks and trait questionnaires. However, the explorative nature of the current study warrants replication. Finally, we outline some scenario aspects that can be further improved, including better physical engagement and standardization.

## Introduction

Aggressive behavior has a significant impact on society and is a major focus of interventions within forensic psychiatry. Valid methods for measuring the disposition of individuals to react aggressively are indispensable for investigating the effectiveness of psychological and pharmacological aggression interventions and for studying underlying psychological mechanisms. Valid aggression assessment is also relevant for diagnostics and monitoring individual treatment progress in clinical practice.

A much-used clinical distinction is ‘reactive’ versus ‘proactive’ aggression (Haller, 2014). The first type is also known as ‘affective’ or ‘impulsive’ aggression. It is an emotion-driven form of aggression in response to a stimulus that can be perceived as threatening, offensive, or frustrating. It has been historically conceived as impulsive, thoughtless and driven by anger (Anderson and Bushman, 2002). Disproportionate reactive aggression is characteristic of individuals who are highly impulsive and/or have difficulty regulating their emotions. The second type, proactive aggression, refers to intentional acts to earn a reward or to gain dominance over others (Dodge et al., 1997). This type of aggression is also known as ‘instrumental’ aggression and is used to achieve a predetermined goal, such as obtaining valuable possessions or status (Haller, 2014).

Although aggressive behavior itself is often impactful and easy to observe, it is more challenging to objectively measure an individual’s disposition to behave aggressively, that is, aggression proneness. The most commonly used aggression measures have critical shortcomings (Lobbestael, 2015). Self-report scales require adequate self-insight, which may be impaired in forensic patient populations (Krakowski and Czobor, 2012). Observation surveys are challenged by observers needing to assess patients in situations where aggression may occur. This is often not possible as psychiatric wards prioritize de-escalation, and outpatient settings are limited to indirect information sources and observations during therapy.

Another way of measuring aggression is through behavioral paradigms. These paradigms involve observation and registration of aggression following provocation. A well-known example is the Point Subtraction Aggression Paradigm (PSAP; Cherek et al., 2003). In this computer task, participants collect points by pressing a button frequently. At some point, a (fictitious) opponent steals their collected points, after which the participant can (1) continue collecting points, (2) defend their collected points for a brief period of time, or (3) steal points from the opponent. The latter option is considered reflecting aggression. Other commonly used aggression paradigms are (variants of) the Taylor Aggression Paradigm (TAP; Taylor, 1967), also known as the Competitive Reaction Time task (Warburton and Bushman, 2019), in which participants can administer loud tones or shocks to an opponent; the hot sauce paradigm (HSP; Lieberman et al., 1999) that operationalizes aggression as the amount of extremely hot sauce administered to an opponent in a food tasting experiment; or the Voodoo doll task (DeWall et al., 2007) where pins can be inserted into a voodoo doll. While laboratory tasks are praised for their high experimental control and internal validity (Banaji and Crowder, 1989), there is controversy about the degree to which these types of sterile, artificial (computer) tasks/games generalize to aggression in everyday life (Parsons, 2015). An additional problem for aggression paradigms is their need for complex cover stories, usually featuring competition with fictional human opponents in an adjacent laboratory or online, which are frequently disbelieved by participants (Lobbestael, 2015).

Virtual Reality (VR) is a promising technology for triggering and assessing aggression that could improve several of the shortcomings of the existing paradigms. First, VR offers the opportunity for *ecologically valid* scenarios, which resemble real-life social interactive situations where aggression is likely to occur. This can be achieved through the use of (programmed) agents or avatars (controlled by experiment-leader) that interact with participants (Blankendaal et al., 2015), mimicking the essential features of everyday social interaction (Hari and Kujala, 2009). Additionally, the presence of avatars overcomes the problem of participants need to believe cover stories.

A second advantage is that VR potentially offers better external validity for (aggressive) behavior; behavior shown by participants in VR resembles real behavior and can be generalized to it. Several VR paradigms offer participants the possibility to express aggression verbally, within dialogs with avatars (Klein Tuente et al., 2020), or physically, by assaulting an avatar with virtual hands (Lobbestael et al., 2021; Verhoef et al., 2021). This is an advantage compared to most behavioral paradigms, in which the expression of aggression is only possible in a strict frame (e.g., actions are limited to three options as in PSAP) and/or is unusual (e.g., applying hot sauce as in HSP, or aversive sounds as in TAP). Furthermore, it has been shown that VR is able to add an emotional, inhibiting component to the aggressive act itself. For example, in the Virtual Trolley Dilemma, increased emotional responses (electrodermal response) were measured when participants decided to throw a person in front of a tram during a moral dilemma (Patil et al., 2013; Navarrete et al., 2012).

The third advantage is the engaging nature of the VR experience, supported by a sense of presence (e.g., the feeling of ‘being there’; Rebelo et al., 2012). This provides an ideal context for provoking aggression. VR has proven to outperform regular computer games. For example, VR survival horror games are known to successfully heighten perceived and psychophysiological anxious responses compared to regular computer games (Bender and Sung, 2021; Pallavicini et al., 2018). Similarly, Vatsal et al. (2024) showed that VR is more effective in eliciting anger emotions than a non-immersive flatscreen. This potential to elicit anger suggests that VR is possibly suitable for evoking reactive aggression, which is the impulsive, emotionally driven variant of aggression (Dodge and Coie, 1987). Furthermore, it is suggested that present and engaged participants might feel freer to express aggressive behavior in VR compared to real-life roleplaying exercises because they then worry less about the relationship with the assessor/therapist (Klein Tuente et al., 2020). Finally, VR offers the possibility to assess aggression in inmates in situations outside the psychiatric clinic, which likely increases its ecological valence.

Fourth, VR is hypothesized to allow for precise experimental control of the environment, despite the relative complexity of ecologically valid environments (Parsons, 2015). Specifically, VR offers the possibility to provide each participant with a standardized VR experience, including standardized environments and the visual and auditive appearances of avatars and/or agents. Standardization is a key component of psychological assessment because equal circumstances are essential for valid comparisons between subjects (Bohil et al., 2011).

To our knowledge, only two studies have assessed aggression with VR so far. Verhoef et al. (2021) developed provocative interactive VR scenarios for children in a classroom setting and found that these VR scenarios evoked higher levels of aggressive social information processing (Lemerise and Arsenio, 2000; Crick and Dodge, 1994) and better predicted real-life aggression compared to vignettes (Verhoef et al., 2021). Lobbestael and Cima (2021) investigated two VR tasks within a student population, with one VR task triggering reactive aggression by viewing and responding to a cheating and insulting dart player, and the other VR task aiming to trigger proactive aggression where participants could earn extra money by aggressing. They found a positive correlation between aggression displayed in this VR reactive aggression task and self-reported aggression, although no such correlation was found for the proactive task.

Research on provocative VR scenarios is thus still limited, making it worthwhile to design and investigate a diverse pallet of VR scenarios involving different types of triggers, provocation methods, situations and avatars to study which are most suitable for aggression assessment. Several of the situational factors of the General Aggression Model (Anderson and Bushman, 2002) can serve as a useful basis for provocation in aggression assessment. These situational factors include aggressive cues, frustration, provocations, and incentives. First, aggressive cues can activate aggression-related concepts in memory. For example, this could include the appearance of a person or specific cues in the surroundings. Second, frustration can be defined as the obstruction of goal achievement (Anderson and Bushman, 2002). According to the cognitive neo-association theory (Berkowitz, 1989) and the earlier frustration–aggression hypothesis (Dollard et al., 1939), frustration can lead to aggressive behavior, especially reactive aggression. Factors that make it difficult or impossible to complete the task, such as time pressure or complexity, can increase frustration. Interpersonal provocation (Anderson and Bushman, 2002; Berkowitz, 1993), such as insults, intimidation, social aggression, or interference with attempts to achieve an important goal (i.e., frustration caused by a human agent), is thought to be a major factor that can lead to aggression. Many of these provocations can be seen as a form of frustration in which a specific person is identified as the one responsible for the failure to reach a goal. Finally, incentives can be used to induce aggression indirectly by influencing implicit or explicit perceptions of the cost–benefit ratio. This can lead to enhanced planned and instrumental aggression, contributing to frustration and evoking reactive aggression.

To our knowledge, there is no VR aggression assessment scenario specifically designed for and investigated within an adult forensic population. However, the literature suggests that this population might be sensitive to specific triggers and provocations. For example, aggression in (forensic) psychiatry is typically related to Cluster B personality disorders, including antisocial personality disorder (Mancke et al., 2018). Both antisocial behavior and aggression are associated with dominant behavior within social interaction (Mazur and Booth, 1998). Furthermore, higher levels of psychopathy—another highly prevalent diagnosis in forensic settings—are associated with dominant counterreactions after confrontation with dominant role players (Lobbestael et al., 2018). Social dominance and control, therefore, seem interesting triggers for aggression, possibly distinguishing between aggressive and non-aggressive individuals.

Within the current project, ‘Virtual Reality Aggression Assessment’ (VRAA), we first developed standardized, provoking scenarios for VR aggression assessment in an adult (forensic) population. The scenarios were based on the social interactive roleplaying module of VR-platform ‘Social Worlds’, developed by CleVR BV, in which an experimenter controls the voice, emotional expressions and gesture animations of avatars. This module was originally developed for the Virtual Reality Aggression Prevention Training (Klein Tuente et al., 2020). Clinical observations have shown that this module can offer useful exercises for provoking tension and aggression in the context of aggression treatment by using personalized, provocative scenarios.

The primary objective of the present research, consisting of two pilot studies, is to investigate whether aggressive behavior can be elicited by standardized, provoking, social interactive scenarios and to what extent the outcome measures form a valid aggression assessment. The first explorative study was conducted with forensic patients with a known disposition to react aggressively. This group of participants was selected to ensure that a failure of the paradigm to elicit aggressive behavior could be attributed to an inadequacy of the paradigm and not merely reflect a low disposition to react aggressively among the participants. In addition, a follow-up explorative study was conducted with a student population to investigate between-group differences and concurrent validity with aggression questionnaires.

## Methods

### Study 1: a pilot study with aggressive patients

#### Participants

In total, 12 adult male participants (M_Age_ = 36.7, *SD* = 8.7) were recruited from a high-security forensic psychiatric center ‘de Pompekliniek’ (*n* = 6) and an outpatient forensic psychiatric clinic ‘Kairos’ (*n* = 6) located in Nijmegen and Arnhem, the Netherlands. The main inclusion criterion was an above-average or high disposition to react aggressively. This was operationalized as at least five points on the Social Dysfunction and Aggression Scale-9 (SDAS-9, Wistedt et al., 1990), as assessed by trained personnel (M_SDAS-9_ = 10.4, *SD* = 6.9). This threshold was in line with previous research (Smeijers et al., 2017). Inpatients were assessed through direct observation on the wards, while outpatients’ assessments relied on therapist observations and information shared by the participants in therapy. Exclusion criteria included a history of epilepsy, a psychotic disorder, an IQ below 70 (M_IQ_ = 92.1, *SD =* 9.2), direct influence of alcohol or drugs, use of medication primarily aimed at reducing aggression, or any (mental) state that gave an impression of high risk for an adverse event.

The sample size of 12 was based on a statistical rule of thumb for explorative studies without *a priori* information. The number was justified by considerations of feasibility, precision about the mean and variance, and regulatory considerations (Julious, 2005).

#### Virtual reality scenario

The interactive VR scenarios were programmed in ‘unity’ code by CleVR BV (Delft, The Netherlands) and projected with a head-mounted display (HMD, Oculus Rift 1). The VR software was installed on a gaming Laptop (BTO X-Book 17CL73-GTX960). The avatars were operated by the researcher, who initiated avatar movements *via* an operator-tablet (Microsoft Surface Pro) and verbally interacted with the use of a USB multi-pattern condenser microphone (Samson C03U) and voice-morphing software (screaming Bee). Participants heard the avatar’s speech and VR background sounds through headphones with noise protection (3 M Peltor) and could interact with the avatars by speaking out loud. In addition, a controller was used to allow for virtual hand movements within the VR environment. Although interaction with the environment was not possible, this feature was thought to enhance engagement and physical actions from the participants in VR. The participant’s perspective in VR on the VR laptop screen allowing the operator to monitor the session.

The scenarios consisted of a series of successive scenes, each lasting about 1–3 min. Verbal interaction and movements of the avatars were written out in detail in a script to ensure optimal standardization. Occasionally, improvisation by the operator was required to maintain realistic interaction in case of unexpected participant reactions (preserving ecological validity), but simultaneously aimed to return to the script with minimal deviation (preserving standardization). Transitions between scenes were marked by a brief fade to black.

**Figure 1:**
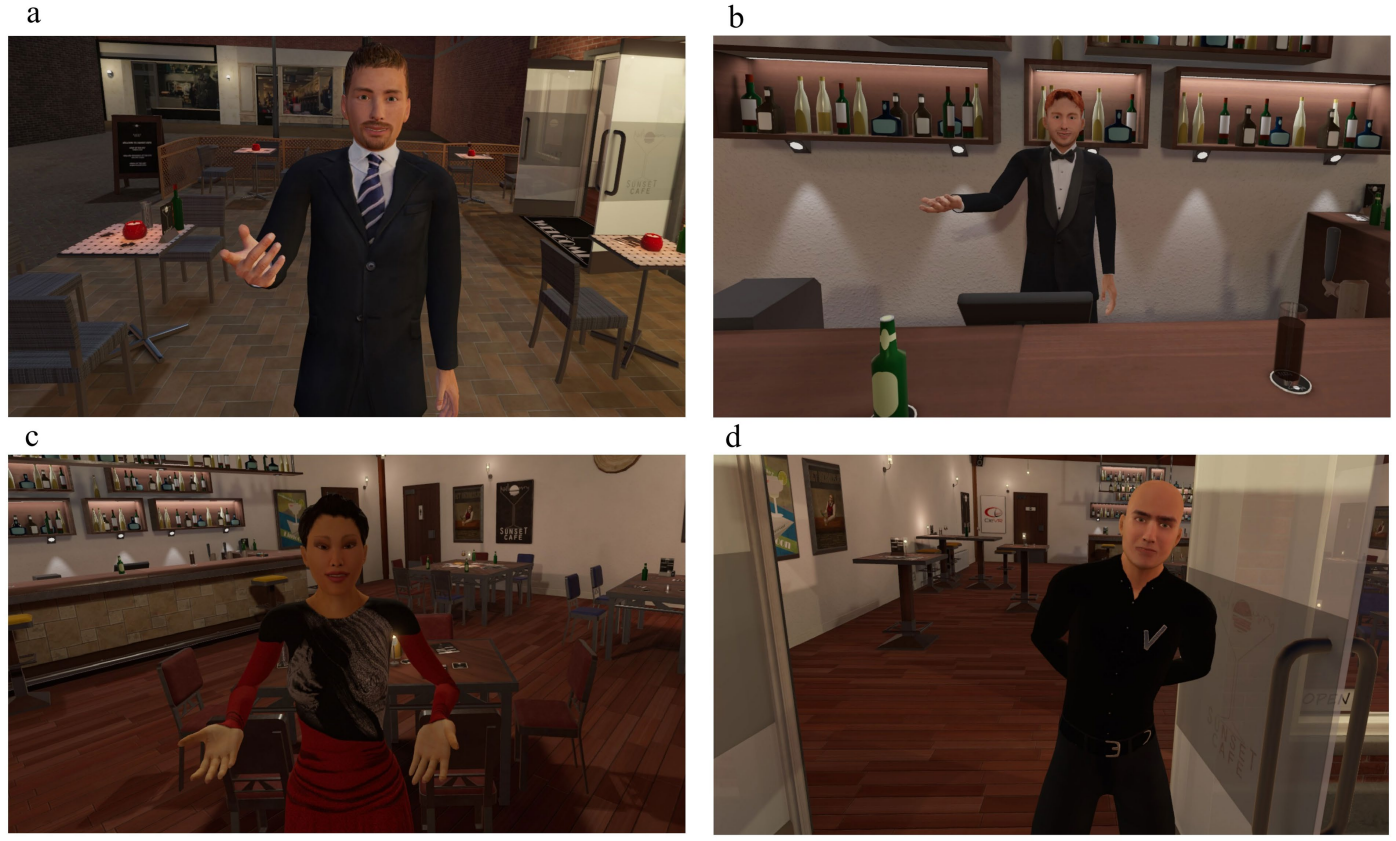
Physical appearances of the avatars in the café scenario. **(a)** Instruction avatar; provides instructions and evaluates performance, **(b)** neutral avatar ‘the barman’; acts cooperative and friendly, **(c)** provocative 1 avatar ‘the lady’; acts uncooperative and socially aggressive, **(d)** provocative 2 avatar ‘the bouncer’; acts socially dominant and disrespectful. The avatars depicted are fictional and do not represent real individuals. ©2025, CleVR. Screenshots used with permission.

Two scenarios were developed for the first study. The first scenario was the café scenario. It consisted of 16 scenes and took place in a café environment (see Figure 1). It was designed to include distinguishable instruction scenes, neutral scenes, and two types of provocative scenes. The scenario began with the instructor avatar welcoming the participant into the VR world and explaining the procedure. The participant had to complete several assignments given by the instructor, for example, asking a neutral barman avatar which beers were on tap. Every successfully accomplished assignment was rewarded with €0,50 as an incentive to be paid after the experiment. Easy-to-accomplish assignments with a neutral, nonprovocative avatar were given first to familiarize the participants with the VR world. Gradually, scenes became more challenging with the introduction of frustration and provocative elements (see Supplementary material 1, for the detailed chronological script).

Frustration was induced in the provocative scenes by adding time pressure and making it difficult, and sometimes impossible, to accomplish assignments. Additionally, financial rewards were withheld and an unexpected financial penalty was given after the instructor censured an assignment as failing (irrespective of the participant’s answer). Provocation was first elicited by interacting with a female avatar called “the lady,” who was noncooperative at first and became verbally offensive and socially aggressive in the second and third scenes. The choice of a female character and this behavior were partly based on forensic psychiatric patients’ descriptions of triggers for intimate partner violence. The second provocative avatar was “the bouncer” who acted very dominantly and unreasonably directly from the outset. Later, he became intimidating and, at some point, threatening with physical aggression. His appearance, tall and wearing a security uniform, served as an aggressive cue. We expected that aggressive behavior would be more frequent and severe during provocative scenes than neutral and instructional scenes.

The second scenario was the so-called ‘street scenario’. However, this scenario will not be discussed further in the present article because the main mechanisms of provocation have failed. This provocative element in this scenario was ‘betrayal’, which involved the participant establishing a companionship with one of the avatars, which later proved to be unreliable. However, most participants failed to establish a companionship with the avatar and unexpectedly indicated that they distrusted the avatar before any provocation occurred. Consequently, little aggression was observed. The street scenario was therefore discontinued after Study 1.

There were two reasons for combining aggressive cues: frustrating elements and provocations. First, it was essential to maximize frustration to answer the question of whether the scenario is capable of eliciting aggression. By incorporating multiple types of provoking situational factors, we increased the likelihood that (1) at least one provocation or frustrative element would resonate, given individuals vary in sensitivity for different triggers, (2) The cumulative effect of multiple aggressive cues, provocations and frustrative elements are likely to increase overall frustration levels, and (3) interactions between these factors could lead to a surplus levels of frustration (e.g., an uncooperative avatar in combination with time pressure is likely to be more provocative than the sum of the individual elements). Second, we consider a mix of provocations to be more ecologically valid since, in real life, aggressive behavior is likely caused by a combination of impellers and instigators (Finkel and Hall, 2018).

We opted for a male avatar in the Provocation 2 scene because male–male aggression is typically the investigated aggression type (Archer, 2004). However, we included a female character in the Provocation 1 scene to offer opportunities for male–female or female–female aggression in psychiatric and general populations, since this is an understudied but relevant topic, for example, in the context of intimate partner violence.

#### Screening measures

The Social Dysfunction and Aggression Scale (SDAS) is a behavioral observation scale for aggressiveness and dysfunctional behavior in the past month (Wistedt et al., 1990). It consists of nine items covering outward aggression (referred to as ‘SDAS-9’) and two items covering inwards aggression. Outward aggression is split into two dimensions: ‘irritations and verbal aggression’ and ‘physical aggression aimed at objects or others’. A total score is calculated from these dimensions. Originally, the questionnaire was developed for clinical observation (Schmand et al., 1992). A 5-point scale ranging from 0 (*not present*) to 4 (*severely present*) is used. Administration time is approximately 5 min. The SDAS has adequate observed reliability, good internal consistency (*α* = 0.79), and convergent validity in comparison with two other outward aggression scales (Wistedt et al., 1990). In the present study, the SDAS-9 is used for screening of the aggression inclusion criteria.

The National Adult Reading Test (Nart-R) is a screenings instrument for IQ consisting of 50 words that increases in level of difficulty (Nelson and Willison, 1991). The NART-R is considered to be a reliable estimate of premorbid ability and shows strong correlations with the WAIS-IV (Bright et al., 2018). In Study 1, the Dutch version of the NART—De Nederlandse Leestijd voor volwassenen (NLV)—was used for the screening of IQ in the context of the exclusion criteria. It shows high correlations with the Verbal IQ (r = 0.85) and Full-Scale IQ (IQ: r = 0.74) and has reliable internal consistency (α = 0.91; Schmand et al., 1992).

#### Primary outcome measure

The Social Aggression and Dysfunction Scale–VRAA (SDAS-VRAA) is based on the SDAS-9 and adapted specifically for the current study to register aggressiveness and dysfunctional social behavior during each VRAA scene. The SDAS-9 was selected as a base because it includes the possibility of scoring relatively mild variations in aggressive behavior, providing a sensitive measure. The social dysfunction items are also relevant because of VRAA’s social interactive aspect.

The wording of the items of the SDAS-9 was adjusted to make it compatible with VR. For example, the behavioral descriptions of ‘*directed verbal/vocal aggressiveness*’ rated on a 5-point scale as follows: *0 = Not present*; *1 = Very slight or doubtful aggressiveness toward the avatar*; *2 = Mild aggressiveness manifested by an explicit way of talking, though the aggressive contents are only present in short outburst*; *3 = Moderate aggressiveness, for example, insulting the avatar personally, more constant sometimes vociferous*; *4 = Severe and sometimes screaming aggressiveness, for example, making serious insults or wishing the avatar harm*.

In contrast to the standard 2-week observation period of the SDAS-9, the SDAS-VRAA was administered directly after every scene. Furthermore, two items about physical aggression were omitted because physical aggression to personnel and objects was not applicable in the scenario. SDAS-VRAA consisted of seven items, with a total score range of 0–28.

#### Trait measures

Lifetime aggression was measured with the Aggression Questionnaire (AQ, Buss and Perry, 1992). It is a self-report questionnaire with 29 items scored on a 5-point Likert scale. “The AQ consists of four factors: Physical Aggression (e.g., *“If somebody hits me, I hit back”*), Verbal Aggression (e.g., *“I cannot help getting into arguments when people disagree with me”*), Anger (e.g., *“I have trouble controlling my temper”*), Hostility (e.g., *“I am suspicious of overly friendly strangers”*).”

Both the total score (*α* = 0.89) and subscale scores (physical aggression, α = 0.85; verbal aggression, α = 0.72; anger, α = 0.83; and hostility, α = 0.77) showed to have good internal consistency. The Dutch version of the AQ was used for administration in Dutch participants, and the overall psychometric qualities were considered good (Morren and Meesters, 2002). Internal consistency was good for the total score (α = 0.86) and physical aggression (α = 0.75), low for verbal aggression (α = 0.51), and moderate for anger (α = 0.67) and hostility (α = 0.69).

The Reactive Proactive Questionnaire (RPQ; Raine et al., 2006) measures reactive aggression (11 items; e.g., “*damaged things because you felt mad*”) and proactive aggression (12 items; “*Hurt others to win a game*”). The items’ answering options are 0 (never), 1 (sometimes) or 2 (often). The English scale shows to have good internal consistency on the reactive (α = 0.84), proactive (α = 0.86) and total aggression scales (α = 0.90). The Dutch version also has good internal consistency for the reactive (α = 0.83), proactive (α = 0.87), and total (α = 0.91) aggression scales (Cima et al., 2013).

#### Interview

A semi-structured interview was conducted directly after both scripts to gain insight about the interpretation of avatars and situations, experienced emotions/distress, ecological validity, presence, recognizability with real-life situations and the effect of the rewards. The main aim of the interview was to find additional, subjective cues for further development of the scenarios and interpretation of observed behavior. The data were not suitable for systematic qualitative analysis or systematic, in-depth discussions.

#### Procedure

The sampling procedures started with a general screening for eligible patients by the treatment coordinators. Candidates were subsequently screened for their disposition to behave aggressively by one of the onsite staff members with the use of the SDAS-9. Inpatients were observed directly in the wards by trained personal, while outpatients’ assessments were based on therapist observations and client information shared during therapy. Staff members asked permission from the candidate to be contacted by the researcher if the criteria were met. The researcher provided an oral and written explanation for the study. Patients had 1 week to decide on participation.

The day of testing started with signing of the informed consent form (10 min), followed by a screening by the researchers for in- and exclusion criteria, including intelligence with the ZAV. Subsequently, the Dutch versions of the AQ and RPQ were administered (20 min). The participant was reminded again of the voluntary characteristic of the study and that VR could be stopped at any moment with no further consequences. The head-mounted display, headphones, and controllers were installed (5 min). The VR scenario started, and the participant completed the café scenario (25 min). One researcher was responsible for running the VR scenario, while the other observed the participant’s behavior and filled out the SDAS-VRAA after each scene. Sessions were videotaped to reassess if necessary, but this was rarely needed.

Participants were reimbursed €15,00 for participation plus the additional rewards (€0.50) for accomplishing the in-game assignments. One week later, the participant was contacted again and asked about any adverse effects. The study has been approved by the medical ethical committee of Radboud UMC (CMO Arnhem – Nijmegen, file number 2018–4426).

### Study 2: a pilot study with students

#### Participants

In total, 12 students (M = 23.92, *SD* = 1.93) of Radboud University Nijmegen participated. Both male (50%) and female (50%) were included. The sample consisted of German (75%) and Dutch (25%) students, all of whom were fluent in English. The same exclusion criteria of Study 1 were applied.

The rationale for the sample size was to match the number of participants with the first study. The research proposal was approved by the ethics committee of the Faculty of Social Sciences (ECSW) of the Radboud University (file number: ECSW-2021-022).

#### Virtual reality scenario

The same VR software, technologies and operating procedures as in Study 1 were used. The HMD was upgraded to Oculus Rift version S. Compared to Study 1, Scene 14 (the third encounter with the lady) was left out, since the 13 scenes before that seemed to be sufficient for provocation, and Scene 14 turned out to be somewhat awkward and irrelevant.

The financial incentives were replaced with points in Study 2. The instructor’s avatar communicated how well they were performing compared to other participants, in order to give the points more meaning. This change was made because the interview results of Study 1 suggested that the (low) financial incentive did not contribute to higher motivation or commitment to accomplish the task for any of the participants, while it was considered being an impractical type of reward for future implementation.

#### Primary outcome measures and trait measures

As in Study 1, the SDAS-VRAA was administered for every scene in VR as a primary outcome of aggression, administered by one of the two researchers. The English versions of the AQ and RPQ were administered to measure the disposition to act aggressively for all participants (see Study 1).

#### Presence and simulator sickness questionnaires

Presence was measured with the use of I-group questionnaire (IPQ; Schubert et al., 2001). It is a self-report questionnaire with 14 items, scored on a 7-point Likert scale ranging from −3 to 3. It aims to measure general presence, spatial presence, involvement, and sense of reality in VR. It is considered having good reliability and validity (Vasconcelos-Raposo et al., 2016).

The 14-item Simulator Sickness Questionnaire (SSQ; Kennedy et al., 1993) measures the degree to which participants experience physical sensations such as nausea, fatigue, and headache. The SSQ is scored on a 4-point scale, ranging from 0 (none) to 3 (severe). While it is generally considered a reliable and sensitive measure, its construct validity and factor structure in the context of VR are currently under debate (Sevinc and Berkman, 2020).

#### Procedure

The participants were recruited using the Radboud University research recruitment website ‘SONA’ and *via* the distribution of the information letter in the personal network of the researchers. Participants had at least 1 week to decide to participate after receiving the information letter. On the day of the experiment, they were informed (again) about what the study involved and about the risks and exclusion criteria. Finally, they signed the informed consent.

Next, the participants were asked to fill out the English versions of the AQ and RPQ (20 min). All questionnaires were filled out on a laptop using a data management platform, Castor EDC. Subsequently, the participants were instructed on how to install the VR glasses and headphones themselves, in order to enter VR and take into account COVID measures (10 min). They started with a non-provocative anxiety assessment scenario (10 min), in which participants had to ask questions to strangers on the street (unrelated to the present study). After a 15-min break, the participants continued in the café scenario (25 min). The scenario was presented in Dutch for Dutch students and in English for non-Dutch students. The scenario was operated by the same researcher as in study one. Afterward, the state subscale of the State Trait Anxiety Inventory (Spielberger et al., 1983), the IPQ, and the SSQ, were administered. Finally, the researchers had a short interview with the participants and a debriefing. Researchers called the participants to check for any adverse effects 1 week after the experiment.

### Statistical analysis

#### Main statistical analysis (within-subject comparisons)

Friedman ANOVAS were used for within-subject comparisons of the dependent variable ‘SDAS-VRAA scores’ of the first 13 scenes of the café scenario. The independent variable ‘Type of scenes’ consisted of instruction (instructor avatar), neutral (barman avatar), and Provocation 1 (lady avatar) and Provocation 2 scenes (bouncer avatar). The Wilcoxon signed-rank tests were used for focused comparisons. Non-parametric tests were chosen because assumptions of heterogeneity and normal distribution were violated due to zero-inflated data of the instruction and neutral groups. A two-tailed *p*-value of 0.05 was maintained as a significance threshold.

#### Explorative statistical analysis (between-subject comparisons and correlations)

Explorative data analyses were conducted by combining the data of both studies. This was possible because the circumstances within the first 13 scenes of the two VR scenario data sets were kept constant (the environment, avatars, script, and operator), and the same trait questionnaires were applied. However, interpretation of these analyses should account for procedural and contextual variations, in part due to the studies being conducted at different times.

Spearman correlations between averaged SDAS-VRAA scores of provocative 1 and 2 scenes, and AQ, and RPQ were calculated for the student and patient data separately. Non-parametric tests were chosen because assumptions of normal distribution were not met, and small sample sizes were used. Subsequently, Spearman correlations were used to analyze the combined student and patient data. Although assumptions of heterogeneity and normality were met, Spearman correlations were chosen for consistency with the correlational analyses of the separate groups.

A two-tailed p-value of 0.05 was maintained as a significance threshold. We considered corrections for multiple comparisons to be overly conservative for the explorative purposes of the current study.

#### Dropout

One patient in Study 1 dropped out in the 12th scene (fifth instructor scene). The provided reason of the participant was that he believed that the scenario was a test for him to act proactively and prevent any further provocative situations by stopping the paradigm. Data on this subject were taken into the analyses because of the explorative nature of the study. The analysis was systematically checked by rerunning all analyses without this subject. This did not alter the decisions about the 0-hypothesis.

## Results

### Main results

#### Type of scene comparisons of study 1

**Figure 2:**
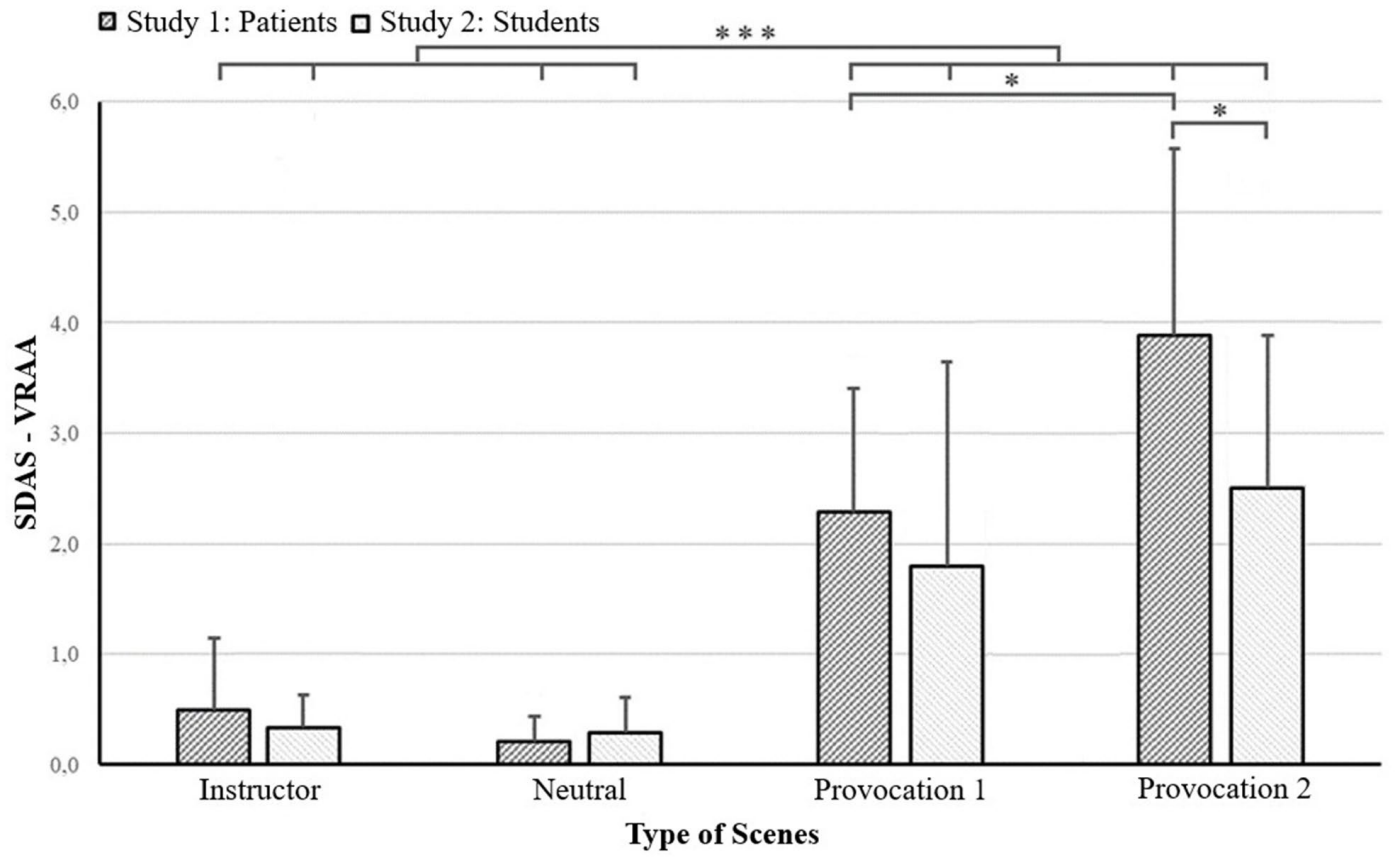
Observed aggression for type of scenes between aggression prone patients and students. *p < 0.05, ***p < 0.001.

The SDAS-VRAA scores of instruction, neutral, Provocation 1 and 2 scenes within the first 13 scenes of the café scenario were compared with the use of a Friedman ANOVA test (Figure 2). Results revealed that the SDAS-VRAA scores differed significantly between the types of scenes (*X^2^*_F_ (3) = 28.71, *p* < 0.001). Focused comparisons (Wilcoxon signed-rank test) showed that SDAS-VRAA scores in the Provocation 1 scenes were significantly higher than both neutral (*Z* = 3.07, *p* < 0.01) and instruction (*Z* = 3.06, *p* < 0.01) scenes. The SDAS-VRAA scores of Provocation 2 scenes were significantly higher than both neutral (*Z* = 3.06, *p* < 0.01) and instruction (*Z* = 2.98, *p* < 0.01) scenes. SDAS-VRAA scores for Provocation 2 were significantly higher than Provocation 1 (*Z* = 2.04, *p* < 0.05). There was no significant difference between neutral and instruction scenes (*Z* = 1.36, *p* > 0.05).

#### Type of scene comparisons of study 2

The SDAS-VRAA scores of instruction, neutral, Provocation 1 and 2 scenes of the first 13 scenes of the café scenario were compared with the use of a Friedman test (Figure 2). Results revealed that average SDAS-VRAA scores differed significantly between the type of scenes (*X^2^*_F_ (3) = 21.69, *p* < 0.01). Focused comparisons (Wilcoxon signed-rank task) showed that SDAS-VRAA scores in the Provocation 1 scenes were significantly higher than both neutral (*Z* = 2.58, *p* < 0.05) and instruction scenes (*Z* = 2.58, *p* < 0.05). SDAS-VRAA of Provocation 2 scenes were significantly higher than both neutral (*Z* = 2.94, *p* < 0.01) and instruction scenes (*Z* = 3.06, *p* < 0.01). No significant difference between neutral and instruction scenes was found (*Z* = 0.97, *p* > 0.05). In contrast with the patient study, no significant difference between Provocation 1 and Provocation 2 scenes (*Z* = 1.65, *p* > 0.05) was found within the student-study.

### Explorative results

#### Between-group comparisons

Comparisons between the patients and students are visualized in Figure 2. Patients and students did not significantly differ in SDAS-VRAA scores in the instruction scenes (Mann–Whitney *U* = 75.50, *p* > 0.05), neutral scenes (Mann–Whitney *U* = 63.00, *p* > 0.05), or Provocation 1 scenes (Mann–Whitney *U* = 93.00, *p* > 0.05). Patients did show a significantly higher SDAS-VRAA score within the Provocation 2 conditions than students (Mann–Whitney *U* = 110.00, *p* < 0.05).

#### Correlational analysis with aggression questionnaires

Table 1 shows the descriptive of the AQ and RPQ and their correlations with the four types of scenes of the café scenario. A significant moderate positive correlation was found between SDAS-VRAA scores of Provocation 2 and the Total AQ. Total SDAS-VRAA scores of the Provocation 2 scene showed significant moderate positive correlations with subscales AQ-physical and AQ-hostile. SDAS-VRAA scores also showed a significant, strong, positive correlation with the RPQ-proactive and a positive, moderate correlation with the RPQ total.

**Table 1:** Aggression questionnaires: descriptives and correlations with SDAS-VRAA of provocative scenes.

Trait aggression questionnaires	Descriptives				Correlations					
	Patients		Students		Patients		Students		Combined	
					Prov1	Prov2	Prov1	Prov2	Prov1	Prov2
	*M (n = 12)*	*SD*	*M (n = 12)*	*SD*	*r (n = 12)*	*r (n = 12)*	*r (n = 12)*	*r (n = 12)*	*r (n = 24)*	*r (n = 24)*
AQ-total	85.1	12.8	76.6	17	0.31	0.19	0.28	0.31	0.35	0.49*
AQ-physical	28.7	5.9	20.1	6.3	−0.06	−0.01	0.10	0.35	0.22	0.46*
AQ-verbal	13.4	3.9	17.5	3.7	0.24	0.06	0.54	0.01	0.20	−0.19
AQ-anger	17.4	3.8	19.2	7.1	0.39	0.10	−0.01	0.52	0.06	0.26
AQ-hostility	25.6	2.6	19.7	5.4	0.56	−0.12	0.22	0.29	0.46*	0.43*
RPQ-total	18.2	11.1	11.2	5.8	−0.08	0.26	0.37	0.19	0.18	0.45*
RPQ-rea	12.1	5.6	9.0	4.0	−0.18	0.15	0.45	0.07	0.19	0.33
RPQ-proa	6.2	6.3	2.2	2.5	−0.04	0.34	0.11	0.41	0.12	0.61**

SDAS-VRAA, Social Dysfunction and Aggression Scale for VR Aggression Assessment (observed aggression in VR); AQ, Aggression Questionnaire (trait aggression); RPQ, Reactive Proactive Questionnaire (trait aggression); M, mean; SD, standard deviation; r, Spearman correlation coefficient; *p < 0.05; **p < 0.01.

No significant correlations were found in the separate analyses for students and patients. This is likely the result of the relatively small sample sizes. SDAS-VRAA scores for both Provocation 1 and 2 scenes, across both patients and students, generally showed weak to moderate non-significant correlations with AQ-total and RPQ-total scores, with the exception of patient SDAS-VRAA scores in Provocation 1, which showed no correlation with RPQ-total. Several moderate correlations were observed, though none reached statistical significance. In patients, Provocation 1 correlated with AQ-anger (*r* = 0.39) and AQ-hostility (*r* = 0.56), and Provocation 2 with RPQ-proactive (*r* = 0.34). In students, Provocation 1 correlated with AQ-Verbal (*r* = 0.54) and RPQ-reactive (*r* = 0.45), and Provocation 2 with AQ-physical (*r* = 0.35), AQ-anger (*r* = 0.52), and RPQ-proactive (*r* = 0.41).

## Discussion

### Main findings

The primary objective of the present study was to explore whether aggression can be elicited and measured within a provoking and frustrating VR scenario called VRAA. The following main conclusions can be drawn. First, results show that in both aggression-prone patients and a student sample, provocative scenes elicited higher aggressive responses than neutral and instructional scenes. This shows that VRAA is capable of evoking aggressive behavior in both populations. These results also suggest that observed aggression is genuinely a response to the provocative interactions, rather than the result of strategic gameplay or random explorative behavior.

The main analysis also showed that the provocative scene with a dominant, unreasonable, intimidating male character elicited more aggression than a non-cooperative, socially aggressive female character within an aggressive patient population. This implies that not every provocation type has the same provocative effect within an aggressive population. This finding aligns with the notion that forensic male patients aim to acquire social dominance in the male-dominated environment of a forensic clinic (Holley et al., 2021). The findings could also be explained by the female gender of the second provocateur, as meta-analyses show that the tendency of subjects to aggress against men is greater than against females (Eagly and Steffen, 1986).

### Between-group differences

Explorative comparisons with combined data from Studies 1 and 2 suggest that the provocative scene with a dominant, unreasonable, intimidating male character elicited more aggression in patients than it did in students. This result is the first indication that this VRAA scene can discriminate between an aggressive and a non-aggressive population. No group differences were found in the provocative scene with a non-cooperative, socially aggressive female character. This implies that it is important to ‘push the right buttons’ and that not every provocation type has the same provocative effect for each group.

There are several possible explanations for the observed difference, though a comprehensive discussion is beyond the scope of this article. One explanation is the previously mentioned tendency of aggressive forensic patients to assert dominant status (Holley et al., 2021), which may be less pronounced in non-aggressive students. Another explanation relates to gender, as Study 2 included a gender-balanced group. Given that males are generally more aggressive than females (Archer, 2004; Eagly and Steffen, 1986), females in the student sample may have contributed to a lower overall average level of aggression in that group. Additionally, the interaction between participant and avatar gender may have influenced responses; for example, female participants may be less likely to aggress against male avatars due to the perceived risk of physical retaliation.

### VR observed aggression and trait aggression

Based on the combined data analyses of Studies 1 and 2, observed aggression in the provocative scene with a dominant, unreasonable, intimidating male character correlated positively with total scores of the trait aggression questionnaires. These results are an indication that observed VR aggression relates to self-rated aggression, supporting our VR scene’s convergent validity. This is in line with another aggression-triggering VR paradigm developed by Lobbestael et al. (2021). This is an interesting finding since literature shows that behavior in some laboratory aggression paradigms rarely correlates with self-reported levels of aggression (Lobbestael, 2015; see also Casula et al., 2023 for a recent study with a lack of correlations between TAP and RPQ, and a non-significant trend of negative correlations in Asaoka et al., 2021 between PSAP and AQ and RPQ). The current results, together with results found by Lobbestael et al. (2021), suggest that provocative VR scenarios are potentially closing the gap between behaviorally assessed aggression and self-rated aggression. The correlations found imply that observed aggression in VRAA generalizes to real-life aggression, suggesting external validity.

When looking at the different types of trait aggression, we first see that observed aggression in the provocative 2 scenes correlates with self-reported physical trait aggression. One explanation is that the Provocation 2 scenes can be seen as a prelude for possible male-to-male physical aggression (a fight with the bouncer), which is not the case for the Provocation 1 scene. It is plausible that lower trait physical aggression individuals avoided further escalation, while, in contrast, individuals with high-physical trait aggression were less avoidant and more prone to escalate with (non-physical) aggression, likely to stay in control by winning the so-called dominance battle. The findings align with dominance being associated with antisocial behavior and aggression (Lobbestael et al., 2018; Mazur and Booth, 1998) and fit the profile of forensic patients who are diagnosed with an antisocial personality disorder. The correlational findings may also be (partially) explained by the subgroup of female students. Females tend to report lower levels on both trait physical aggression questionnaires and behavior tasks (Archer, 2004).

Furthermore, self-reported hostility correlated with observed aggression of both provocative avatars in the combined analyses. Here, hostility was operationalized as hostile interpretation bias—the tendency to perceive and interpret social information in a hostile manner (Smeijers et al., 2022). The found correlation aligns with the literature indicating that hostility biases are often associated with aggression-prone individuals in both the general population and clinical population (Bushman, 2016), in particular in relation to reactive aggression (Dodge, 2006; Smeijers et al., 2019).

A third finding related to specific types of aggression is that VR-observed aggression correlated with the trait of proactive aggression. This again aligns with our VR scenario involving social dominance gain provided in Scene 2 since proactive aggression reflects “intentional acts to earn a reward or *to gain dominance over others*” (Dodge et al., 1997). It is also in line with a recent study evidencing that both the proactive RPQ subscale correlated with a dominance subscale of the aggressiveness questionnaire (Dinić and Raine, 2019), albeit that the study also found a link with the reactive RPQ scale, which was not the case in our study.

No significant correlations were found in the separate analyses for students and patients, likely due to the small sample sizes. The non-significant correlation coefficients may suggest an overall trend of weak-to-medium correlations of observed aggression with trait questionnaires’ total scores, although still weaker than those found in the combined sample. This suggests that correlations of the combined data are partly driven by between-group differences and/or increased variance. Additionally, there is no clear evidence for differences in correlation strength between the aggressive and non-aggressive groups. The subscale items show more diffuse trends, with several specific cases of strong correlations. However, these findings remain inconclusive and require replication with larger samples.

### VRAA compared to existing aggression paradigms

VRAA appears to have several advantages compared to existing laboratory aggression assessment paradigms. First, VR environments of VRAA give the impression of better ecological validity. A main contributor to this is the social interactive component, which is a typical situation in which real-life aggression occurs. A second advantage is that the interaction with the avatar was so engaging that it was not necessary to pretend that the avatars were operated by other human players, solving the problem with cover stories. A third benefit is that the scenarios offer a suitable platform for ecologically valid observations beyond aggression alone, as the VR also offers the opportunity to observe, for example, participants` interaction skills, coping mechanisms, or social strategies. Thoughts, interpretations, and emotions could also be addressed directly after the VR experience in the semi-structured interview. This additional information can be relevant to explore the underlying psychological mechanisms of individual patients in clinical practice. A disadvantage compared to existing (non-VR) laboratory paradigms is that VRAA is more complex to administer than existing paradigms, mainly because it requires more technological and roleplaying skills of the operator. Furthermore, the equipment is more expensive. Finally, it can be argued that the VR scenario offers less precise experimental control than the existing paradigms. This will be discussed in more detail in the following paragraph.

### Ecological validity versus experimental control

In general, the levels of experimental control in VR were found to be sufficient to establish the present favorable validity results. This supports the hypothesis that VR is able to feature both ecological validity and experimental control, offering the best of both worlds (Parsons, 2015). However, two dilemmas in the progress of developing the scenario illustrate that the relationship between ecological validity and experimental control can be conflicting at some points.

First, while we choose to combine multiple provocations, opting for a pure or single provocation would likely have less variance/error, which could positively impact the replicability of the results. Second, VRAA made use of live and flexible control of avatars by the operator, which kept dialogs realistic in favor of ecological validity, but arguably at the cost of some degree of standardization. It would, therefore, be interesting to investigate an alternative VR paradigm with pre-recorded spoken text of the avatars (e.g., Verhoef et al., 2021). The current study thus prioritized ecological validity. This is also beneficial for clinical applications, as it offers more ability for free observations. In contrast, when one’s main focus is on group comparisons, experimental control should be prioritized.

### Recommendations for future VR-scenario development

Several recommendations for the development of VR scenarios can be formulated. First, VRAA offers only limited possibilities for physical interaction by the participant. Therefore, it is recommended that future scenarios provide participants with the ability to manipulate objects, turn virtual hands into fists, and activate automatic avatar responses and sound effects when touched. Incorporating these features potentially increases the likelihood of physical aggression.

Second, participants indicated in the interview that external incentives (both a small amount of money and a ranking list) were not very important to them. In future research, improved commitment could still be sought in different ways of external motivation. However, it will remain difficult to replicate the levels of commitment found in real-life situations, where usually much more is at stake. A potentially more interesting way to increase commitment lies within intrinsic motivation by increasing the attractiveness of playing and accomplishing the tasks themselves.

Third, the levels of observed aggression are, in general, defined as ‘lightly present’ and ‘mild’ rather than ‘average’ or ‘serious’ according to scale-item definitions. This raises the question of whether we should aim for higher levels of aggression in future VR designs. On the one hand, higher aggression levels could result in more variance in outcomes, which could be beneficial for correlational research and comparisons between conditions and groups. On the other hand, too severe or uncontrolled levels of aggression are not desirable because of safety and ethical reasons. The current results show that mild levels of evoked aggression are, in principle, sufficient for valid aggression assessment, suggesting that aiming for (much) higher levels might not be necessary.

Fourth, while the results are not conclusive, the absence of unrealistic high aggression may indicate that VRAA is generalizable to real life, that is, it has external validity. Unrealistic high levels of aggression would be a sign of applying game strategy or simply trying things out. This applies to students but also to forensic patients, who have the capability and motivation to control their behavior due to the forensic psychiatric setting and because they could mentally prepare for the experiment.

Finally, we think that generalizability is worthwhile to set as a target for future scenario development since it is a unique feature of VR. The better the evoked behavior within VR resembles real-life behavior, the more relevant the tool becomes for clinical practice. It offers the possibility to not only predict aggressive behavior but also to observe what the behavior looks like and to address processes and psychological mechanisms leading to this behavior. The key to improving the external validity of scenarios lies in the proper representation of typical social situations that are meaningful for aggressive patients.

## Limitations and recommendations for future research

A main limitation of the current study is that data from Studies 1 and 2 were combined for exploratory analyses. While the VR scenarios were identical, offering a unique opportunity for analysis between an aggressive patient sample and a non-aggressive student sample, there were differences in procedures and contextual factors, partly because studies were conducted at different times. The following main alternative explanations for between-group differences should be taken into account. First, it could be that observers differed in their observation skills, despite the well-defined observational items and training. Note that this also had a methodological benefit because both observers were blind to the reactions of the other subgroup, diminishing observer bias. Second, translation from Dutch to English might have resulted in either increased or decreased provocative effects. Third, the non-provocative scenario anxiety could have affected aggression. Fourth, a newer version of the HMD has been used in Study 2. Finally, COVID (rules) might have affected student behavior, though we consider it unlikely that these would have specifically affected provoked aggression. Given these potential confounders, the datasets are not strictly comparable. However, we consider the comparisons still informative for exploratory purposes in the context of future scenario development and research.

A second limitation of this study is the low number of included participants. Analyses of between-group comparisons and correlations, but not within-subject comparisons, could have been underpowered, resulting in insensitivity to weaker or moderate effects. This applies in particular to the correlational analyses within separate groups. Also, a larger sample size would allow a more detailed investigation of subgroup characteristics. Future research could explore how different patient populations with varying psychopathologies interact with VRAA. This would also allow for more conclusive insights into gender effects, which are particularly interesting given the potential for gender-based interaction effects between avatars and participants.

The present study primarily investigates whether VR can be used to evoke and measure aggression in general. The next step could be gaining a deeper understanding of how the VR scenario evokes aggression and investigating which specific provocations and psychological mechanisms contribute to these scenarios. Additionally, it can be interesting to investigate the unique contributions of VR to the scenario. Such questions could be addressed by including systematic, qualitative analyses of semi-structured participant interviews, or experimentally manipulated provocations for an effective scenario.

Taking these limitations into account, the conclusions of this pioneering and exploratory study should be regarded as a proof of concept that VR can be used for aggression triggering and assessment. It offers directions for further VR scenario development, follow-up research, and a broader discussion on the use of VR in (aggression) assessment.

## Conclusion

The current study shows that aggressive behavior can be evoked with our VR aggression assessment scenario (VRAA) and that the level of aggression can be systematically assessed by a standardized aggression observation scale. Explorative results suggest that VRAA possesses construct, concurrent and known-group validity. Future research with adapted VR scenarios will provide further insights into the possible psychometric advantages of the VR approach.

## Data Availability

The raw data supporting the conclusions of this article will be made available by the authors, without undue reservation.
