# Supplementary material for: Virtual reality aggression assessment with social interaction: early evidence for validity from two pilot studies
Source: Front Psychol. 2025 Jun 13;16:1585609. doi: 10.3389/fpsyg.2025.1585609 (PMC12202414; doi:10.3389/fpsyg.2025.1585609)
Supplement: Supplementary file 1 [file Supplementary_file_1.docx]

**Supplement 1**

*Description of scenes in the café scenario*

| **Scene** | **Avatar** | **Type** | **Content** |  |
| --- | --- | --- | --- | --- |
| 1 | Instructor | Instruction | Gives assignment: What time does the café open? |  |
| 2 | Barman | Neutral | Is cooperative and makes friendly conversation. |  |
| 3 | Instructor | Instruction | Gives feedback on positive result and gives assignment: What kind of beer is on draft? Time pressure is added. |  |
| 4 | Barman | Neutral | Is cooperative and friendly. |  |
| 5 | Instructor | Instruction | Gives feedback on positive result and gives new assignment: How much do the beers cost? |  |
| 6 | Lady | Provocative / Frustrating | Is not cooperative, complaining and does not give room for participant to speak, is offending towards participant later (tension is building up) |  |
| 7 | Barman | Neutral | Is cooperative and friendly |  |
| 8 | Instructor | Instruction | Gives Feedback: correct answer but too slow. Instructor is dominant and says that he is disappointed. Next assignment: which whiskey do they have? |  |
| 9 | Bouncer | Provocative | Acts dominantly: Demands to spell name, and to turn around (to check for weapons and drugs). Is framing the participant negatively. Tells that participant can go in but has to be quick. | |
| 10 | Lady | Provocative | Not cooperative. Is saying negative things about participant: mainly that he/she is very boring. Social aggression (saying that she is going to gossip with the bouncer). Shows middle finger | |
| 11 | Bouncer | Provocative | Is angry. He is calling the participant a liar and he/she is bothering the lady. He is making a step forward. He is making some serious threats. | |
| 12 | Instructor | Instruction | Says that the goal is not achieved, but that it does not influence the rating because it was a difficult scene.  Encourages to try the assignment one more time. | |
| 13 | Bouncer | Provocative | At first again threatening, but lets the participant through in the end. | |
| 14* | Lady | Provocative | Looks scared, but still behaves provocative. Finally she says that the barman has arrived. | |
| 15 | Barman | Neutral | Cooperative and friendly | |
| 16 | Instructor | Instruction | Says that the participant performed very well and tells that this is the last scene. | |

*This scene was left out in study 2.
